# Asian ageing: The relationship between the elderly population and economic growth in the Asian context

**DOI:** 10.1371/journal.pone.0284895

**Published:** 2023-04-24

**Authors:** Thaveesha Jayawardhana, Sachini Anuththara, Thamasha Nimnadi, Ridhmi Karadanaarachchi, Ruwan Jayathilaka, Kethaka Galappaththi

**Affiliations:** 1 Sri Lanka Institute of Information Technology, SLIIT Business School, Malabe, Sri Lanka; 2 Department of Information Management, Sri Lanka Institute of Information Technology, SLIIT Business School, Malabe, Sri Lanka; Southeast University, BANGLADESH

## Abstract

The elderly population and economic growth have been a contentious topic among researchers. Regardless of the economic growth rate, the population and its growth have a stimulating influence on economic development. This study aims to explore the relationship between the elderly population and economic growth in 15 Asian countries, based on secondary data gathered from the WDI (World Development Indicators) from 1961 to 2021. This research contributes to filling the empirical gap, capturing the Granger causality concerning the relationship between the elderly population and economic growth in the Asian context in a single study. The empirical findings highlighted a one-way Granger causality from economic growth to the elderly population for India, Japan, Malaysia, and Singapore while vice versa for Bangladesh, China, and Pakistan. Furthermore, for Nepal, there is a two-way Granger causality, while there is no Granger causality for remaining countries. To the best of the authors’ knowledge, this study has been the first to investigate the relationship between the elderly population and economic growth for Asian nations, using a lengthy data series and a Granger causality test. The main findings will assist the governments, policymakers, and foreign investors in effective decision-making in this regard.

## 1. Introduction

The elderly population of a country can either be a blessing or a burden [1]. The ADB [2] showed that by 2030, the Asian continent would be the region with the largest elderly population in the world, exceeding 4.9 billion (Bn). With the advancement in technology, extended healthcare services, and accessibility to high level of assisted care facilities, the aged population can impact on the economy in varying magnitudes and hence, is worth analysing. This study focuses primarily on how the elderly population in Asian countries has impacted economic growth and vice versa.

Lin and Wang [3] highlighted that the rise of the elderly population, in the long run, provides a comparably substantial burden to society, especially in the Asian region. On the one hand, this is due to Asian cultural beliefs like multigenerational relationships, close-knit families, living arrangements etc., compared to those in other regions. It is where living of three to four generations is common in the same household. Therefore, Huang, Lin [4] stated that the rise of the ageing population may impact economic growth. Still, its overall association with the long-term economic growth of a country has to be measured in the social, cultural as well as the political context. On the other hand, several research studies have revealed a positive relationship between the elderly population and economic growth [5–7]. Many studies discuss the elderly population and economic growth associated with macroeconomic factors such as savings rate, public expenditure, and the spillover effect. However, the current study primarily narrowed down the research investigating whether the elderly population has a clear association with the country’s economic growth.

The objective of this paper is to identify the relationship between economic growth and ageing population in Asian countries. Cross-country empirical studies investigating the relationship between ageing populations and economic growth in the Asian region are limited. As such, this research differs from existing studies to date and contributes to the literature in three ways.

Firstly, this research study covers numerous countries in the Asian continent, with the latest data collected over a long period. Only a very few studies have been conducted so far for the Asian region, even for a short period. Thus, this study is unique in terms of the extent to which the countries and years are covered. Secondly, a country-wise analysis has been conducted for all the countries considered, which would allow the researchers to compare the behaviour of the two variables among various countries. The study findings provide valuable insights into the effectiveness of the policies implemented by various governments in the past years. Finally, the economic boom experienced over the past few decades by most Asian countries resulted from a large portion of working-aged people contributing to the economy. Most of them have reached or are reaching their old age at present. Findings in this study would be beneficial for such nations to implement policies to get the aged to continue contributing to the national economies or propose initiatives where there is a tendency to do so.

The rest of the paper is structured in the following manner. Section 2 presents a review of previous studies on the association between the elderly population and economic growth. The data and methodology are detailed in Section 3. The empirical results and their interpretations are presented in Section 4, and the study concludes with policy implications in Section 5.

## 2. Literature review

By 2030, the global ageing population, particularly in Asia, is expected to reach at least 30% of the total population [8]. While in contrast, this may seem like a less convincing figure, as when the overall view is considered, it showcases severe economic concerns and a considerable issue that must be dealt with. One of the main points analysed for the Asian economy is the high rise in the Asian economy after 2003, with countries such as India, Singapore, Indonesia, China, and Taiwan leading the way to economic growth [9]. According to Lin and Wang [3], the rising population of middle-income earners, tech bubble burst, and the extended life expectancy have contributed to a marked increase in societal growth over time.

Even though the growing proportion of elderly people in the population may not enhance economic performance in the short term, it may contribute to economic growth in the long run. This could be attributed to the accumulation of capital and assets, and the consumption patterns of the elderly in developing nations. Specifically, the increase in life expectancy and age of retirement may improve the employability of elderly workers, hence increasing their household income and consumption. Therefore, the elderly population has a favourable impact on economic growth according to findings of previous scholars [10–14]. Nevertheless, literature exists with opposing results across regions and countries worldwide. In those studies, the negative effect of elderly population on economic growth have been mainly attributed to the rising elderly care costs to the government and the burden of elderly on the working population [15–17]. Thus, an interesting and ongoing debate is clearly visible among research community regarding the relationship between the elderly population and economic growth.

In terms of the South Asian region, India and Bangladesh have a record high of over 8% of the ageing population in the region. However, as Huang, Lin [4] showed, this ageing population and their impact on the economy is relatively lower considering that they have a solid support network. This finding illustrates the likelihood of a shallow impact on the economy considering the household base work and unpaid home tasks being conducted by the aged population. As Deloitte [8] highlighted, in the South Asian region, many aged populations still live with their parents, meaning more than one generation lives in the same household. This is not only a phenomenon in India but also in Bangladesh, Pakistan, and even in Sri Lanka. Henceforth, this shows that in South Asia, the burden placed on the ageing population is much less due to the overall economic gain that they provide through housework and child rearing.

China’s economic growth rate during the past three decades was among the highest globally [18]. However, recent trends indicate that the population of China is ageing. The demographic structure started to change since the one-child policy was put into place in the 1970s: the dependency ratio for children is falling, while the dependency ratio for the elderly is rising [19]. Further, in terms of China, the ageing population tends to have a severe case in terms of the burden on economic growth, due to the one-child policy the Chinese have followed in the last four decades. The productive population of society has declined by 12%, incurring an additional burden on the working population because they themselves need to take care of their aged parents [3]. Moreover, the Huang, Lin [4] emphasised the Chinese context’s need for investments and savings for the elderly. Therefore, when Chinese parents age, they tend to have a very strong financial asset portfolio to fall back on and the means to support their children through higher education, and sometimes through early years of career growth [3]. Thus, the Chinese ageing population does tend to have a minimal effect on the disposable incomes of the individual working population, and yet they need to be prepared with a strong fallback plan for the later years. The assets grown over the years are all passed out to the next generation, which has been a major positive attribute of the Chinese population.

One of the Asian region’s heightened growth potentials relies on Southeast Asia, where it showcases more than 67% of the ageing population. This is where even the population above 60 continually works within the society, primarily on some entrepreneurial ventures, especially in countries such as Thailand, Taiwan, and Indonesia, where the ageing population continues to work past their prime years. Countries such as Japan tend to have a very high orientation towards work, and this is illustrated by their focus on having a high level of life expectation [20]. According to Yang, Zheng [9], at present, Japan has the highest life expectancy in the Asian region, well past the average of 72 years. On top of this, in some areas such as Okinawa, it is common to find individuals past 80 years are still actively contributing to the economy.

Over 6%–15% of the ageing population is evident in the volatility of Asian societies, as [21] highlighted, the impact of the ageing population on economic growth is relatively low. This is acceptable considering the fact that people have a very strong focus on keeping with the Asian values of living with parents, which eases the burden of the aged individuals on the economy. The main point of analysis here is that, unlike in the Western economies, the burden of the ageing population is quite low in the Asian economies. As noted before, three generations of families living together in the same household while the aged population is taking care of children, is a very common attribute identified in the current Asian society. While Western values have been inflicted on the Asian society, Yang, Zheng [9] showed the rational context of living with parents. The high savings rate and keeping child rearing centred around home have helped create a strong economic fallback plan for Asia without shouldering the economic burden of the aged population.

It is well known that the elderly population in East Asian countries plays a vital role in economic prosperity. In contrast, the working elderly population in this region exceeds the working younger population [22]. Moving towards the economic status in Asia, tourism and financial development boost the economic growth of Asia, where the work of the elderly well-experienced population has significantly contributed towards the economic development [23]. Many researchers have examined the association between the elderly population and economic growth country wise and region wise. For instance, Salman and Zaib [24] investigated the association among dependency and the saving rate over the period 1980–2009 regarding the Pakistan economy using Pearson correlation and multiple regression methods. Results indicated a negative relationship between the dependency ratio with savings and the population of Pakistan has evolved over the past years; this showed a greater emphasis towards the increasing dependency of the elderly on younger generations [24]. Authentication suggests that active participation of the elderly people has boosted economic growth. In a similar study, Feng, Cramm [25] examined the longitudinal link between income and social participation among the elderly population in China by acquiring data from China Health and Retirement Longitudinal Study (CHARLS). This study was conducted among people aged above 45 years during the period 2011–2012. The study results revealed that the elderly people in China generating higher levels of household income are positively correlated towards their social contribution. Similarly, Li, Li [26] also investigated how demographic shift can affect the economies of East Asian countries by analysing Chinese provincial level data in the period 1985–2005 utilising the Generalised Method of Moments (GMM). They discovered that the elderly population has a positive impact towards the savings rate, revealing that old people has made a significant contribution towards Chinese economic growth. This scenario has already been discussed/confirmed by Wei and Hao [27] taking provincial data during the period 1989–2004.

Macroeconomic performance in Asia, and especially East Asia, is closely monitored by variations in age structure brought on by demographic shifts, according to Bloom, Canning [28]. Further, they mentioned that the rise in the working-age share supported by a decrease in the youth-age shares, assisted in raising income in the past. Kelley and Schmidt [29], Ge, Yang [30] have also previously discussed these theoretical explanations, which aided in conducting this particular study carried out by Bloom, Canning [28]. Several studies have focused on the impact of the age structure of the population on economic development. This has also been examined in the context of China by Zhang, Zhang [31] by using panel data of 28 Chinese provinces for the period 1990–2005. Their analysis demonstrated that internal changes in the demographic makeup of the working elderly population play a significant role and almost 19% of the working elderly population has contributed to the increase of gross domestic product (GDP) between the period of 1990–2005. A similar approach has been adopted by Bils and Klenow [32]; Ge, Yang [30] in investigating the economic implications of the demographic age structure by emphasising on working old population. Moreover, several research studies seek to look into the relationship between demographics and overall household savings [33,34]. Warburton, Ng [35] showed the impact of social and cultural changes on economic crisis in East Asian countries concerning retirement. The said paper emphasised on the link between the elderly population and economic growth in the Asian context from 1961 to 2021 using the Granger Causality test, which is lacking in previous studies.

According to conventional economic theory, an ageing population may decelerate/slow down the economy. An ageing population is a factor that can dampen economic expansion, according to the life cycle theory advanced by Modigliani [36]. The life cycle theory predicts that a country’s savings rate will grow as its population ages. Although this hypothesis predicts a decrease in aggregate savings as the population ages and a greater percentage of the population reaches retirement age, it also indicates that the proportion of the population reaching retirement age will grow.

In addition, according to Solow’s growth theory, an ageing population makes it harder for a nation to maintain steady economic growth. If the population’s age distribution is constant/stays the same, it can be assumed that the economic growth is stable or the economy is steady. Nonetheless, the age structure of an economy is unpredictable as the population ages. As such, this scenario is practicable only when the economy moves closer to its stable state. According to this view, a country’s economy would suffer if its population continues to age [37].

According to Mohd, Ishak [38], the rise in life expectancy in Malaysia has resulted in higher public expenditures on retirement benefits, healthcare, and the Employees’ Provident Fund (EPF). Since people live longer, there are more people in the senior age group, which has repercussions for the economy and society as a whole. Because of this, people’s wallets and government coffers will all feel the pinch. In such a scenario, providing necessities like shelter and medical care comes at a high cost. In addition, Louria [39] stressed that a rise in the national average life expectancy could extend human life to between 100 and 120 years. Due to this, several societal problems may arise, including higher healthcare costs for the elderly population, lower standards of living/impaired quality of life, increased demand for public services, and difficulties in securing retirement and social benefits. Baharin and Saad [40], using the autoregressive distributed lag (ARDL) approach and a vector error correction model, corroborated these results showing that the ageing population has had a major impact on healthcare costs in Malaysia. Ismail, Abd Rahman [41] provided the reduced old-age dependency may result in lower tax and social security contributions paid by employed people to fund retirement income and health care for the elderly, which may increase labor supply. This results in fewer mouths to feed and more disposable income that can be invested in the economy’s growth. As a result, a rise in the old age dependence ratio indicates a rise in ageing population, which in turn reduces productivity.

Rašticová, Birčiaková [42] show that there is a significant relationship exists between economic activity and health status. Workers who are economically active tend to be in better health status than those who are not. The authors suggest that this may be because work provides social contact, a sense of purpose, and a regular routine, which can have a positive impact on health. The authors also examine the role of age management in the workplace. They argue that age management practices, such as training and development programs, flexible work arrangements, and health promotion initiatives, can help to support the economic activity and health of older workers. The authors suggest that employers should adopt age management practices to ensure that older workers can continue working in a healthy and productive way. Gorzeń-Mitka, Sipa [43] discover that age management is a complex and multidimensional issue that requires a holistic approach. They identify several dimensions of age management, including workforce planning, recruitment and selection, training and development, health and safety, work-life balance, and retirement and post-retirement policies. The authors also highlight the importance of age diversity in the workplace, and the benefits it can bring to organisations. Moreover, they explain that age diversity can lead to increased creativity, innovation, and problem-solving ability, as well as improved organisational performance and productivity. However, the authors also acknowledge that age diversity can also present challenges, such as generational differences in values, attitudes, and work styles, and potential age discrimination.

Many empirical studies have examined the ageing population’s impact on economic growth, with contradictory findings. Studies have identified a negative correlation between an ageing population and economic growth, and one such research was by Brendan and Sek [44]. They used an ARDL bound testing strategy and discovered an important/ a critical negative long-run link between reliance on the elderly and GDP growth in India. This is especially true for India, which faces the prospect of a decline in saving rates and an increase in unemployment as its population ages. Most nations, including South Korea, the Philippines, Thailand, Malaysia, and Singapore, showed a negative impact from relying on the old age dependency ratio, although the findings were statistically insignificant. The dropping birth rate and increasing ageing proxies (such as the old age dependency ratio and the population aged 65+) have dampened economic development in other research by Maestas, Mullen [45]. Teixeira, Renuga Nagarajan [46] observed that although an ageing population may slow/dampen the economic development of high-income nations, it has no such effect on the expansion of low- and middle-income states. They claimed that developing nations’ development prospects are highly diminished by an increase in the old age dependence ratio, and that less developed countries’ growth is severely constrained by an increase in both the ageing index and the old age dependency ratio.

In contrast, research studies have shown a positive correlation between ageing population and economic expansion, as per Brendan and Sek [44]. According to their research, Japan is well advanced into the ageing period since it has the biggest reliance among the elderly. Japan has been implementing active ageing policies (such as the Silver Human Resource Centre) and other policies that encourage the inclusion of the elderly in the workforce to address these issues. These efforts will help mitigate the harmful effects of ageing and generate economic benefits. Ismail, Abd Rahman [41] and Taasim [47] used ARDL in their research and discovered that the elderly population, the elderly dependence ratio, and lower fertility contribute to economic development. Population ageing benefits economic growth, according to Futagami and Nakajima [48], who cited innovations in labour-saving technologies and more expenditures in human capital as examples. Scarth [49] contended that an ageing population could boost productivity by encouraging more investment in human capital. This is because workers will soon be a scarce production element.

This research questions about the relationship between the elderly population and economic growth. Furthermore, the objective of this paper is to identify the relationship between economic growth and ageing population in Asian countries. As a result, this study differs from earlier research and adds to the body of knowledge in three ways.

To begin, this research study spans several Asian countries, using the most recent data collected over a long period of time. Even for a brief period, very few studies have been conducted thus far for the Asian region. In light of how extensively the countries and years are covered, this study is exceptional. Furthermore, a country-wise analysis has been conducted for all the countries considered. Accordingly, this research contributes to filling the empirical causality by capturing the Granger causality concerning the research problem. Granger causality is a method for determining the relationship between two variables. "Causality" is related to the cause and effect concept, but it is not the same. It is a statistical concept based on forecasting.

Although there is sufficient literature on the relationship between the elderly population and economic growth, it is known that the relationship changes as a result of short-term and medium-term policy implementations as well as other macroeconomic conditions. As a result, the model developed in this study fills a gap in the literature that has yet to be filled.

## 3. Methodology

This study was reviewed and approved by Sri Lanka Institute of Information Technology (SLIIT) Business School and the SLIIT ethical review board. Study used the secondary data sources and the data file used for the study is presented in S1 Appendix. This study adopted a quantitative approach based on time-series data analysis of secondary data gathered from the WDI (World Development Indicators) online database. Economic growth was measured by the GDP growth rate (as an annual percentage) in each nation using constant local currency, allowing cross-country comparisons. In addition, the elderly population was measured using the population aged 65 and older (as a percentage of the total population). For a more comprehensive data analysis, annual data were collected from 1961 to 2021 for 15 Asian countries.

This section reveals the research method, the method of data collection, and the economic model to be followed for conducting this research. The Granger causality approach was utilised for this study. Here, two stationary covariance variables, X and Y, are monitored across t periods. If it can be proved the significant influence of lagged values of a variable X in a regression model of Y that depends not just on X but also on its own lagged values Y_t-1_, Y_t-2_, etc., then it can be argued that X Granger causes Y and potentially Y also Granger causes changes in X [50]. The study objective is to determine whether there is a causal link between the ageing population and economic growth. It is possible that the link between the elderly population and economic development is unidirectional, bidirectional, or that there is no interdependence. The direction of Granger causality between variables is determined through four phases utilising STATA software. Using time series data, the Dickey Fuller (Dfuller) and Phillips–Perron (Pperron) unit root tests assisted in determining the stationary characteristics of the population aged 65 and over and the per capita GDP as the first phase. The Vector Auto Regression (VAR) model may be used for the second phase of the linear Granger causality test if all variables are stationary. The optimal lag length is then determined with the lag selection criteria. The Granger causality test finally evaluates the direction of causality between variables as the final step. The Granger Causality formula is as follows:

$${PGDPGR}_{t}=C_{0}+\;\sum \alpha_{i}\;{PGDPGR}_{t-i}+\;\sum \beta_{i}\;{EPOP}_{t-i}+\;\varepsilon_{t}$$ (1)

$${EPOP}_{t}=C_{0}+\;\sum \alpha_{i}\;{EPOP}_{t-i}+\;\sum \beta_{i}\;{PGDPGR}_{t-i}+\;\varepsilon_{t}$$ (2)

Where the unidirectional causation between elderly population (EPOP) and per capita gross domestic product growth rate (PGDPGR) is stated if the sum of estimated coefficients on lagged EPOP is statistically different from zero and the sum of estimated coefficients on lagged PGDPGR is not statistically different from zero (Eq (1)). If the opposite findings are achieved, Eq (2) specifies the inverse relationship between PGDPGR and EPOP. Gujarati [50] affirmed that if both coefficients are statistically different from zero, bidirectional causation can be seen in which both variables influence one another.

## 4. Empirical results

This section reports the main findings of the empirical approach. First, the summary provides the descriptive statistics, then the results of the unit root test and lag length criteria, respectively. Finally, the results of the Granger causality test are provided.

**Fig 1:**
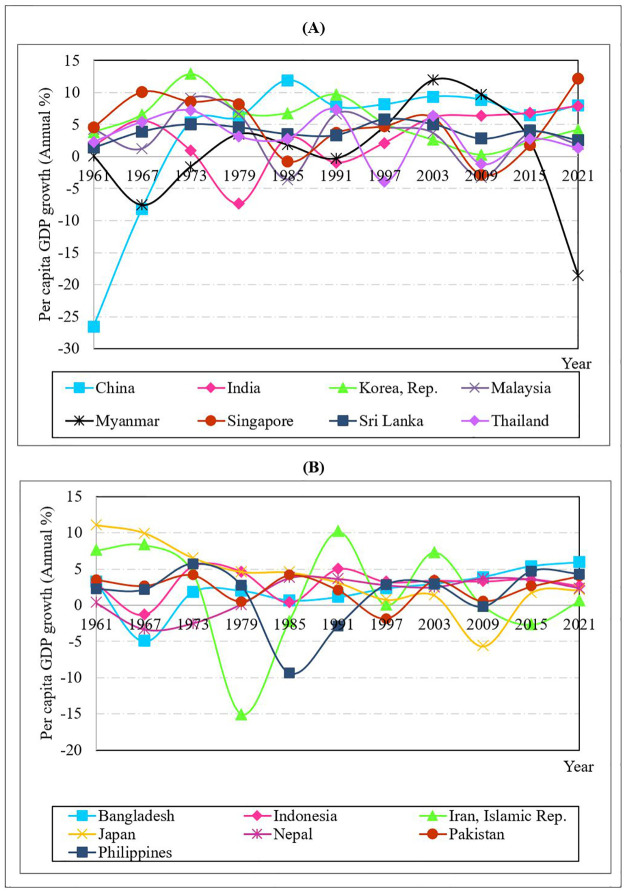
Per capita GDP growth (annual percentage): A comparison. Source: WDI [51].

Fig 1(A) shows the flow of per capita GDP growth rates from 1961 to 2021 in the eight countries with the highest mean per capita GDP growth rates. China, the country with the highest average per capita GDP growth rates, showed rapid growth from 1961 to 1973 because of the Great Leap Forward, and then a slight growth till 2021. Since 1961, the per capita GDP growth rate has fluctuated in all countries owing to the cyclical nature of economic growth. According to Table 1, the countries with the highest average per capita GDP growth rates after China are Korea, Singapore, Thailand, Myanmar, Malaysia, Sri Lanka, and India, respectively. Korea had a high per capita GDP during 1973–1978, while Singapore had the highest per capita GDP in 2021. While the general cyclical nature of economic growth is visible throughout the period for all the countries, a few interesting observations could also be made regarding certain fluctuations. The catastrophic drought resulted in negative economic growth in India around 1979. The Tom Yam Kung crisis that emerged in Thailand in July 1997, which later spread into East Asia and Southeast Asia, was referred to as the Asian financial crisis. The countries most impacted by the crisis include Indonesia, South Korea, Malaysia, and Thailand. Singapore was less impacted than the others, although all were struggling with a decline in consumer demand and lack of confidence across the region. However, this financial crisis saw a speedy recovery in 1999. Myanmar displayed a lower value in 2021 because of the continuing effects of the military coup and the rise of the coronavirus disease (COVID-19) cases resulting in economic meltdown in the same year.

**Table 1:** Descriptive statistics.

Country	Variables	Mean	Sd.	Min.	Max
Bangladesh	PGDP growth rate	2.2440	3.8443	-15.3864	7.8308
	EPOP	3.6542	0.8765	2.6680	5.3308
China	PGDP growth rate	6.7362	6.5248	-26.5276	16.0504
	EPOP	6.2138	2.3185	3.4238	12.4132
India	PGDP growth rate	3.1660	3.3471	-7.5157	7.8999
	EPOP	4.2273	0.9528	3.1002	6.7757
Indonesia	PGDP growth rate	3.1486	3.3098	-14.3506	7.9436
	EPOP	4.2266	0.8711	3.3015	6.5119
Iran	PGDP growth rate	1.7186	7.8878	-24.4661	18.2604
	EPOP	4.0911	1.0911	2.8599	6.8021
Japan	PGDP growth rate	2.9354	3.5790	-5.6815	11.6174
	EPOP	14.4251	7.3121	5.7291	28.6980
Korea	PGDP growth rate	5.9012	3.7881	-5.8118	12.8844
	EPOP	6.8868	3.7618	3.3932	16.5668
Malaysia	PGDP growth rate	3.6797	3.3946	-9.6712	9.1153
	EPOP	4.2050	1.0840	3.1992	7.4455
Myanmar	PGDP growth rate	3.8090	5.6223	-18.5777	12.7224
	EPOP	4.2478	0.7580	3.3555	6.4689
Nepal	PGDP growth rate	1.9676	2.9067	-5.2142	7.5210
	EPOP	3.8577	0.9669	2.6009	5.9109
Pakistan	PGDP growth rate	2.3139	2.2931	-3.2624	8.3966
	EPOP	4.0144	0.1853	3.7912	4.3991
Philippines	PGDP growth rate	1.7940	3.3362	-10.7275	5.8033
	EPOP	3.5153	0.7049	2.9678	5.7218
Singapore	PGDP growth rate	4.9721	4.3321	-5.5551	12.5085
	EPOP	5.9903	2.6499	2.1400	14.2655
Sri Lanka	PGDP growth rate	3.2261	2.3676	-4.1253	9.0029
	EPOP	5.9754	2.1364	3.6846	11.6334
Thailand	PGDP growth rate	4.0374	3.5044	-8.7417	11.3364
	EPOP	6.0342	2.9320	3.3356	13.5357

Note: PGDP denotes the Per Capita Gross Domestic Product, EPOP denotes the Elderly population. The table is based on 61 observations.

Fig 1(B) shows the flow of per capita GDP growth rates from 1961 to 2021 in the 7 countries with the lowest mean per capita GDP growth rates. Fluctuations can be attributable to a string of events with adverse consequences. The Islamic Republic of Iran, the country with the lowest average per capita GDP growth rates, showed a negative slope from 1976 to 1981. The Iranian revolution against the state’s monarchy in 1979 dealt a massive blow to the Iranian economy, nearly doubling the price of crude oil. According to Table 1, the countries with the lowest average per capita GDP growth rate after the Islamic Republic of Iran are the Philippines, Nepal, Bangladesh, Pakistan, Japan, and Indonesia. After experiencing years of growth, the Philippine economy suffered a downturn between 1973 and 1986 due to the Marcos family’s economic plunder. Apart from these, Bangladesh experienced a setback in 1971 due to the Bangladesh Liberation War. However, from 1981 to 2021, a comparatively significant rise in economic growth was seen in Bangladesh. From 2003 to 2011, Japan’s economy showed a downward trend compared to other countries due to factors such as rising debt levels, the global financial crisis, the tsunami, and the nuclear disaster. Nevertheless, even though some countries, such as Japan and Iran, have low per capita GDP growth rates, their economies are still managing well due to national per capita GDP being sizeable in absolute terms.

**Fig 2:**
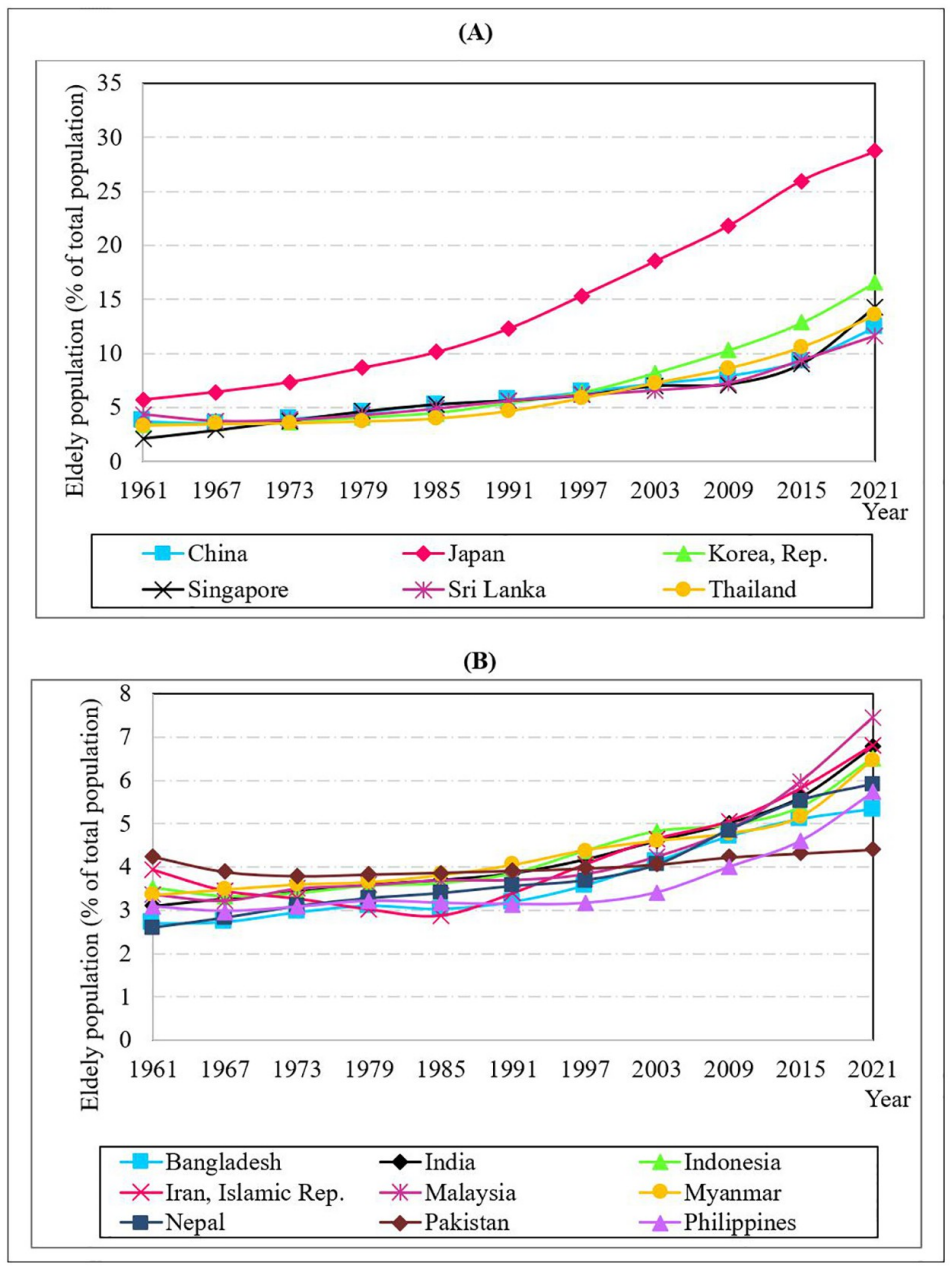
Population aged 65 and older (as a percentage of the total population): A comparison. Source: WDI [51].

Fig 2(A) and 2(B) depict the flow of the elderly population above 65 growth rates from 1961 to 2021 in the countries with the highest and lowest mean elderly population growth rates, respectively, based on Table 1. Overall, the elderly population steadily increased during the 61 years in all countries. From 1961 to 2021, Japan had the highest mean elderly population growth rates, with the most significant increase among all countries. High longevity and low fertility have been attributed to a large proportion of elders in Japan. Even Korean Republic displays a much higher increase in the elderly population as a proportion of the total in recent years due to less than the global average fertility rate. However, Pakistan, on the other hand, was able to maintain a nearly equal proportion of the elderly population over the years with a steady fertility rate.

### 4.1 Unit root test

Prior to the Granger causality analysis, researchers tested for the stationarity of the per capita GDP growth rate and the elderly population. Accordingly, the Dfuller unit root test was developed by David Dickey and Wayne Fuller in 1979 and the Pperron unit root test was introduced by Peter C. B. Phillips and Pierre Perron in 1988 were employed in the study. The null hypothesis of the Dfuller test is that there is a unit root, which implies that the data series is not stationary. The alternative hypothesis is generally stationarity or trend stationarity but can vary depending on the version of the test being used. The null hypothesis of the Pperron test is that the variable contains a unit root, and the alternative is that a stationary process generated the variable. S2 Appendix reports the results of the time series unit root test suggested by Dickey and Fuller for the elderly population (percentage of the total population) and per capita GDP growth (annual percentage). S3 Appendix reports the results of the time series unit root test suggested by Phillips and Perron.

Researchers first performed a Dfuller unit root test for the per capita GDP growth rate variable. Except for Myanmar, the per capita GDP variable was stationary at the first level for 14 Asian nations. Myanmar stopped moving after the first per capita GDP growth rate difference (DGDP). The elderly population variable was then subjected to the same test, and the researchers discovered that Pakistan was unit roots in the elderly population’s first difference (DEPOP). At the second difference (DDEPOP), all 14 remaining nations became stationary.

The Pperron unit root test for the per capita GDP growth rate variable was then carried out by researchers as a next step. While Myanmar became stationary at the first difference (DGDP) of per capita GDP growth rate, the per capita GDP growth rate variable was stationary at the first level for 14 remaining countries, similar to the Dfuller test. The Pperron test was then applied to the elderly population variable, and it was discovered that Pakistan has unit roots in the elderly population’s first difference (DEPOP). The second difference in the elderly population (DDEPOP) caused the other 14 countries to become stationary.

### 4.2 Lag length criteria

To carry out the causality test meaningfully, it is important to determine the optimal lag length. To discover the optimal moments and model lag order, lag selection criteria can be leveraged to calculate selection order statistics. First, the appropriate measures are identified. Lag selection criteria report the final prediction error (FPE), Akaike’s information criterion (AIC), Schwarz’s Bayesian information criterion (SBIC), and the Hannan and Quinn information criterion (HQIC) lag order selection statistics. Akaike’s FPE criterion provides a measure of model quality by simulating the situation where the model is tested on a different data set. The optimal lag length in the VAR specification is determined based on AIC, SBIC, and HQIC. According to Liew [52], the optimal lag length should be the one that minimises the SBIC, AIC, and HQIC information criteria. When mixed results are obtained, the decision is based on the minimum value in the AIC criterion.

According to S4 Appendix, the default maximum number of lags is 10. In Indonesia, Malaysia, and Philippines, the AIC, HQIC, and SBIC criteria are lower when selecting zero lags. Selecting one lag for China, Korea, and Thailand, lowering the AIC, HQIC, and SBIC criteria. Further, the AIC and HQIC criteria are lower when selecting one lag for Iran and Sri Lanka. But the SBIC criterion is lower when selecting zero lags. Selecting seven lags for Pakistan and Singapore lowers the AIC criterion in our case. Here, selecting ten lags for Bangladesh, eight lags for India, two lags for Japan and nine lags for Myanmar lowers the AIC criterion. In Nepal, the HQIC and SBIC criteria are lower when selecting zero lags, but the AIC criterion is lower when selecting five lags.

**Fig 3:**
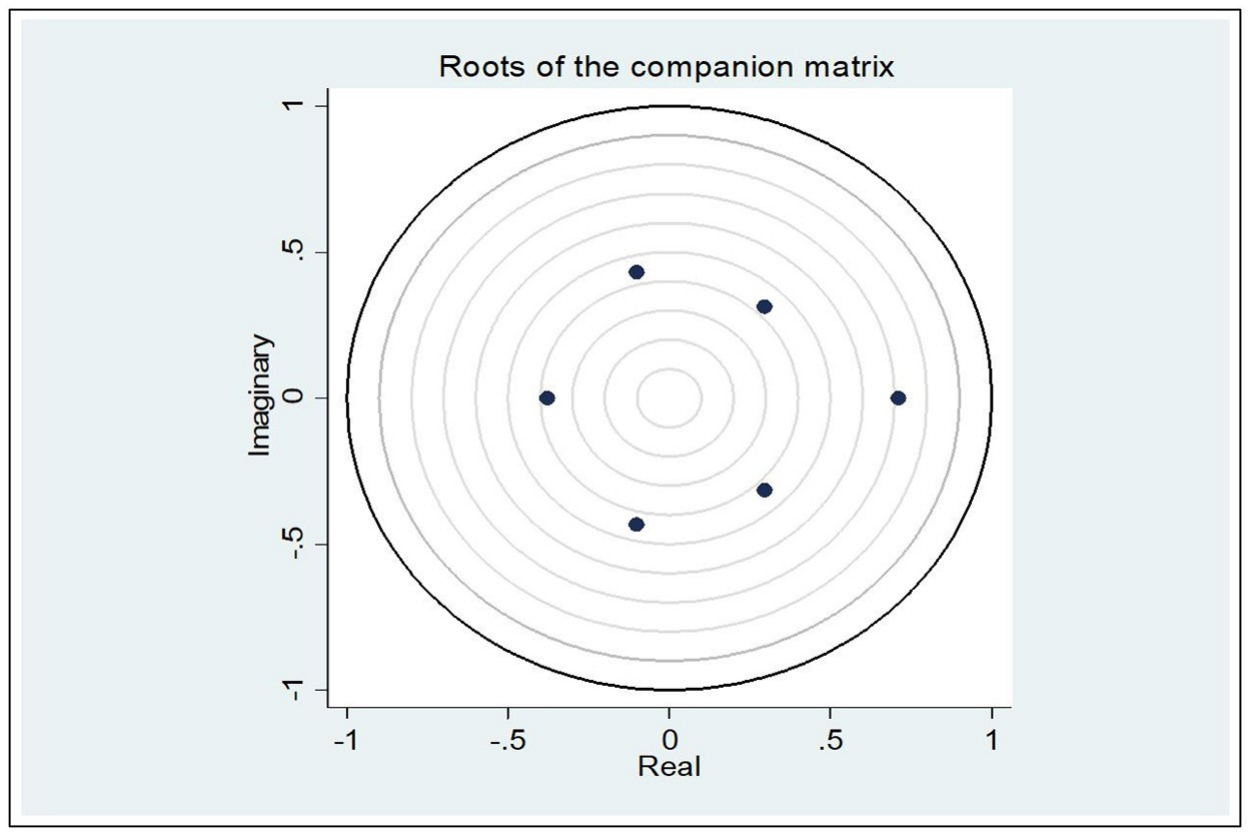
Stability graph.

Stationarity and stability are two crucial presumptions regarding the data variables and the system as a whole that support the validity of multivariate and bivariate modelling in terms of the VAR model. The employed variables’ stationarity has already been established. According to formal definitions, the VAR model is stable if all the coefficient matrix’s eigenvalues are less than 1 [53]. Because all of the eigenvalues in our example are contained within the unit circle (Fig 3), VAR satisfies the stability criterion.

### 4.3 Granger causality test

The Granger causality approach was used to examine the structures of the causal relationships between variables. The Granger causality test is a statistical hypothesis test used to determine whether one time series can forecast another.

The results of the Granger causality test are reported in Table 2. Generally, there is reasonable evidence that the elderly population and economic growth Granger cause each other, except for Indonesia, Iran, Korea, Myanmar, Philippines, Sri Lanka, and Thailand. The findings for Thailand, Indonesia, and Sri Lanka are consistent with those of Dawson and Tiffin [54] and Chang, Chu [55]. They discovered that elderly population growth neither Granger causes economic growth nor is caused by the latter. In other words, the growing elderly population neither stimulates nor detracts economic growth.

**Table 2:** Granger causality test results.

	**DGDP → DDEPOP**	**DDEPOP→DGDP**
Myanmar	0.4520	1.2374
	**GDP → DEPOP**	**DEPOP → GDP**
Pakistan	3.4293	6.4875*
	**GDP → DDEPOP**	**DDEPOP → GDP**
Bangladesh	0.1298	10.267**
China	2.5588	10.19**
India	8.9898**	0.2241
Indonesia	2.6763	0.6209
Iran	0.9923	0.8736
Japan	14.968***	3.1440
Korea	5.7353	5.8787
Malaysia	7.2133*	0.3171
Nepal	8.3927**	7.3877*
Philippines	2.2765	0.7762
Singapore	8.6138**	1.0221
Sri Lanka	1.8467	1.5250
Thailand	2.3449	1.5482

Notes:

*, denotes significance at the 10% level,

**, denotes significance at the 5% level,

***, denotes significance at the 1% level.

DGDP = first difference of GDP; DDEPOP = second difference of elderly population.

For Nepal, there is bidirectional causality between the elderly population and economic growth, which contradicts the results of Dawson and Tiffin [54] and Thornton [56]. The reason could be that this study has used the latest data available for Nepal, thus, reflecting the effects from recent steps taken by the Government of Nepal towards wellbeing of senior citizens in terms of financial assistance and local community and business planning.

In Bangladesh, China, and Pakistan, elderly population growth is found to Granger cause the economic growth. In India, Japan, Malaysia, and Singapore, economic growth is found to Granger cause of the elderly population growth. Table 3 additionally presents the outcomes of the Granger causality test. As a result, the relationship between the elderly population and economic growth is complex. There is no compelling evidence that an ageing population will boost economic growth. Furthermore, the relationship between the elderly population and economic growth differs across countries with similar levels of development.

**Table 3:** Granger causality test results.

Country	GDP → EPOP
Bangladesh	one-way (←)
China	one-way (←)
India	one-way (→)
Indonesia	no-way (↮)
Iran	no-way (↮)
Japan	one-way (→)
Korea	no-way (↮)
Malaysia	one-way (→)
Myanmar	no-way (↮)
Nepal	two-way (↔)
Pakistan	one-way (←)
Philippines	no-way (↮)
Singapore	one-way (→)
Sri Lanka	no-way (↮)
Thailand	no-way (↮)

On the one hand, elderly population growth may be beneficial or detrimental to economic growth, and economic growth may influence elderly population growth. As a result, some Asian economies that achieve low levels of economic growth may not be affected by elderly population growth but rather by other factors such as political instability and a lack of investment. On the other hand, some Asian economies that achieved high rates of economic growth may have done so, not because of elderly population growth but because of other factors, according to Tsen and Furuoka [57]. Non-directionality proves that while demography is an important variable related to economic growth, it is not the only influencing factor on economic growth and vice versa.

Results confirm the presence of multiple factors which need to be considered when analysing the elderly population growth and the impact on economic growth. As noted before, the relationship between these two variables tends to be much more complex, when analysing countries with high population density, such as China, which is predicted to reach the highest economic growth in 2030 in Asia [13]. Hence, the country will beside have a substantially higher elderly population. However, this will be offset by the increase in birth rates and the high productivity yield of its citizens, as more than 70% of this elderly population work in the informal economy after retirement. The same scenario can be applied to countries such as Japan, where the state-of-the-art medical benefits, healthcare technology advancements, and the greatest lifestyle habits have gifted these residents a higher life expectancy. Still, they are economically active even after 65 years of age [9]. Hence while cyclical economic changes can impact the economic growth cycle in Pakistan, Malaysia, Sri Lanka, and Iran, the country-specific issues can be a significant detrimental factor to economic growth more than the rise of an elderly population [1]. Thus, issues hidden underneath the visible factors, such as population ageing would be interesting to analyse in future research. In other words, it is worth exploring beyond just the tip of the iceberg to unveil underlying root causes to gain a holistic picture.

Lin and Wang [3] highlighted the importance of identifying these complicated factors differently, even before the pandemic. While the Asian region had the highest growth potential for the next decade, the occurrence of specific issues such as high government reliance and intervention for economic growth, higher debt supporting major infrastructure facilities, strong geopolitical stresses dismantling the domestic political stability of the countries showed the vulnerability of the economic structures of these countries. This situation is discernible in countries such as Pakistan, Sri Lanka, and even China, as in the current context [4]. Therefore, according to Yang, Zheng [9], while the elderly population may be impacted to a certain extent by the growth of the per capita GDP, it is minuscule and difficult to isolate as a single factor when considering the complexity of the current global economic changes experienced after 2020. It is vital that the progression from post pandemic era, the change in demographic construction of countries in Asia should be identified and revived in order to face the challenges that Asia may have in future. Nonetheless, insights provided by this study will be valuable to governments and institutions to have at least a general understanding of the nature of the relationship between the elderly population and economic growth.

## 5. Conclusion and policy implication

This paper has summarised the results of the relationship between the elderly population and economic growth in the Asian region. Key actors like governments, development organisations, human rights organisations, and charities in the Asian region could better understand the elderly population and economy through this study to give priority to the elderly and countries’ economies. In addition, the study can assist to identify ways to lessen and manage the risks that the elderly population and government face (e.g., environmental assessment, healthcare policy, retirement regulation, city development code, and demographic distribution).

The results of the Dfuller and PPerron unit root test statistics show that, generally, the elderly population are non-stationary at levels one and two, but becomes stationary after taking the second difference. The economic growth is stationary at the first level except for Myanmar.

The lag selection criteria show that the majority of the criteria chose a lag length between one and zero as the optimal lag length. Therefore, all subsequent analyses will be performed using the optimal lag length of this result.

Furthermore, the study estimates the Granger causality between the elderly population and economic growth. On the one hand, for Nepal, there is bidirectional Granger causality between the elderly population and economic growth. For Bangladesh, China, and Pakistan, the elderly population is found to Granger cause economic growth but not vice versa. On the other hand, for India, Japan, Malaysia, and Singapore, economic growth is found to Granger cause elderly population but not vice versa. For Indonesia, Iran, Korea, Myanmar, Philippines, Sri Lanka, and Thailand, there is no evidence of Granger causality between the elderly population and economic growth.

The relationship between the elderly population and economic growth is not definitive. On the one hand, elderly population growth may be beneficial or detrimental to economic growth, and on the other hand, economic growth may influence elderly population growth.

Considering the elderly population is on the rise and there being certain limitations on the resource definition, the following policy implementation will be much more suitable for the Asian continent. According to Deloitte [8], an inherent advantage is evident in the growth of the elderly population, but this must be strategically managed.

Primarily, countries with a high share of elderly population should focus on structural reform policies that do not push up the cost of the elderly population and pass the burden to the government. In particular, the ADB [2] positioned this debate by stating that the rising pension expenses for the government could destabilise the economy, similar to the scenario in the 2009 Greece recession. Therefore, structural policies should be formed with the idea of improving the country’s economic growth and macroeconomic integrity and improving a flexible labour market that can adapt to the changes in the economy and market forces [1]. Nevertheless, elderly care must be assured without the government bearing the whole burden but by spreading it among organisations, such as charities and insurance companies.

The second policy reform for the elderly population in the more demographically congested countries is to focus on reorientating the special protection systems towards alleviating poverty. This can be primarily through creating a strong intergenerational flow of wealth, fiscal sustainability, and social security measures [8]. The report stressed that the wealth building pension schemes, allowing the generations to flow their wealth within minimum tax concessions, can have greater concerns regarding this issue.

The third, population-related policy implications are much more valid for cultures with developing economies, where the focus is on data consensus. Especially, through creating storage systems for data development, policies protecting the vulnerable groups of society, and the continuous analysis of demographical change. With such a data-oriented, evidence-backed strategy, one can really relate to how the elderly population can either be a benefit or a burden. Yang, Zheng [9] highlighted that the lack of tech infrastructure to become a smart society in Asia is the main barrier, which needs to be focused on with very storage micro and macro-economic policies. Better data usage with advanced analytics could help developing nations implement more suitable policies.

The majority of secondary data used in this study were obtained from the WDI. However, Due to the lack of accurate data from 1961 to 2021. Researchers of this study could only obtain data for a few countries. Furthermore, by introducing new concepts and approaches, this study can serve as an initiative to expand future studies’ research strategies to other regions. In this study, we derived these findings using only two variables and as it provides a framework for future research in this field, future researchers will be able to add other demographic and economic variables and carry out further studies. In this regard, including variables such as dependency ratio, savings rates and household income could provide better economic insights. At the same time, moderator variables such as educational level, technological advancement, and quality of life could be useful to understand the relationship between the elderly population and economic growth to a much larger extent. Even at this level, the findings of this study will be helpful during policy implementations by governments and other policymaking bodies since the effectiveness of policies devised for Asian countries in the past could be identified from this research.

## Supporting information

S1 Appendix Data file. (XLSX)

S2 Appendix Dfuller test result. (DOCX)

S3 Appendix Pperron test results. (DOCX)

S4 Appendix Lag length criteria results. (DOCX)
