# Supplementary material for: Asian ageing: The relationship between the elderly population and economic growth in the Asian context
Source: PLoS One. 2023 Apr 24;18(4):e0284895. doi: 10.1371/journal.pone.0284895 (PMC10124889; doi:10.1371/journal.pone.0284895)
Supplement: S2 Appendix — (DOCX) [file pone.0284895.s002.docx]

**S2 Appendix.** **Dickey–Fuller test result**

| **Country** | **GDP** | **DGDP** | **EPOP** | **DEPOP** | **DDEPOP** |
| --- | --- | --- | --- | --- | --- |
| Bangladesh | -6.217*** | - | 3.979 | -1.834 | -6.694*** |
| China | -7.357*** | - | 12.1 | -0.189 | -4.913*** |
| India | -7.005*** | - | 17.339 | 0.473 | -7.783*** |
| Indonesia | -5.431*** | - | 8.5 | 0.017 | -7.443*** |
| Iran | -4.467*** | - | 7.109 | -0.298 | -6.719*** |
| Japan | -4.194*** | - | 10.265 | -1.404 | -2.898** |
| Korea | -5.196*** | - | 36.961 | 4.189 | -4.422*** |
| Malaysia | -6.589*** | - | 18.07 | -0.101 | -6.284*** |
| Myanmar | -1.609 | -5.914*** | 10.319 | 0.756 | -6.856*** |
| Nepal | -8.626*** | - | 5.206 | -1.611 | -7.495*** |
| Pakistan | -6.503*** | - | 0.854 | -2.669* | - |
| Philippines | -5.4*** | - | 16.357 | 0.431 | -7.118*** |
| Singapore | -5.7*** | - | 8.253 | 0.862 | -5.492*** |
| Sri Lanka | -5.177*** | - | 11.819 | -0.878 | -6.152*** |
| Thailand | -4.764*** | - | 29.613 | 5.66 | -2.899** |
