# Supplementary material for: Asian ageing: The relationship between the elderly population and economic growth in the Asian context
Source: PLoS One. 2023 Apr 24;18(4):e0284895. doi: 10.1371/journal.pone.0284895 (PMC10124889; doi:10.1371/journal.pone.0284895)
Supplement: S3 Appendix — (DOCX) [file pone.0284895.s003.docx]

**S3 Appendix. Pperron test results**

| **Country** | **GDP** | **DGDP** | **EPOP** | **DEPOP** | **DDEPOP** |
| --- | --- | --- | --- | --- | --- |
| Bangladesh | -6.329*** | - | 2.045 | -1.862 | -6.624*** |
| China | -7.523*** | - | 6.533 | -0.654 | -4.983*** |
| India | -7.061*** | - | 10.647 | 0.936 | -7.85*** |
| Indonesia | -5.419*** | - | 4.631 | 0.117 | -7.438*** |
| Iran | -4.439*** | - | 3.527 | -0.371 | -6.707*** |
| Japan | -4.051*** | - | 5.613 | -1.479 | -2.863** |
| Korea | -5.247*** | - | 21.273 | 3.205 | -4.436*** |
| Malaysia | -6.585*** | - | 11.209 | -0.1 | -6.187*** |
| Myanmar | -1.699 | -5.845*** | 5.531 | 0.799 | -6.846*** |
| Nepal | -8.576*** | - | 2.694 | -1.556 | -7.489*** |
| Pakistan | -6.565*** | - | -0.179 | -2.683* | - |
| Philippines | -5.426*** | - | 9.436 | 0.397 | -7.118*** |
| Singapore | -5.687*** | - | 4.188 | 0.552 | -5.423*** |
| Sri Lanka | -5.188*** | - | 6.501 | -1.056 | -6.176*** |
| Thailand | -4.78*** | - | 15.437 | 3.489 | -2.822* |
