# Supplementary material for: Asian ageing: The relationship between the elderly population and economic growth in the Asian context
Source: PLoS One. 2023 Apr 24;18(4):e0284895. doi: 10.1371/journal.pone.0284895 (PMC10124889; doi:10.1371/journal.pone.0284895)
Supplement: S4 Appendix — (DOCX) [file pone.0284895.s004.docx]

**S4 Appendix. Lag length criteria results**

| Country | Lag | P | FPE | AIC | HQIC | SBIC |
| --- | --- | --- | --- | --- | --- | --- |
| Bangladesh | 0 |  | 0.0022 | -0.4487 | -.419421* | -.3715* |
|  | 1 | 0.739 | 0.0025 | -0.326 | -0.2381 | -0.0943 |
|  | 2 | 0.849 | 0.0028 | -0.1907 | -0.0442 | 0.1954 |
|  | 3 | 0.009 | 0.0025 | -0.301 | -0.096 | 0.2395 |
|  | 4 | 0.193 | 0.0027 | -0.2618 | 0.0018 | 0.4331 |
|  | 5 | 0 | 0.002 | -0.5395 | -0.2173 | 0.3099 |
|  | 6 | 0.221 | 0.0021 | -0.4931 | -0.1122 | 0.5108 |
|  | 7 | 0.033 | 0.0021 | -0.5441 | -0.1047 | 0.6141 |
|  | 8 | 0.017 | 0.0019 | -0.6264 | -0.1284 | 0.6863 |
|  | 9 | 0.682 | 0.0022 | -0.51 | 0.0466 | 0.9571 |
|  | 10 | 0 | .001638* | -.856968* | -0.2418 | 0.7646 |
| China | 0 |  | 0.01069 | 1.13767 | 1.16697 | 1.21489 |
|  | 1 | 0 | .006053* | .568303* | .656191* | .799954* |
|  | 2 | 0.845 | 0.00694 | 0.7031 | 0.84958 | 1.08918 |
|  | 3 | 0.742 | 0.00786 | 0.82621 | 1.03128 | 1.36672 |
|  | 4 | 0.8 | 0.00899 | 0.95582 | 1.21948 | 1.65077 |
|  | 5 | 0.19 | 0.00941 | 0.9941 | 1.31636 | 1.84349 |
|  | 6 | 0.409 | 0.01032 | 1.07619 | 1.45704 | 2.08002 |
|  | 7 | 0.66 | 0.01174 | 1.19023 | 1.62967 | 2.34849 |
|  | 8 | 0.013 | 0.01088 | 1.09455 | 1.59259 | 2.40725 |
|  | 9 | 0.657 | 0.0125 | 1.20815 | 1.76478 | 2.67528 |
|  | 10 | 0.009 | 0.01154 | 1.09517 | 1.71039 | 2.71673 |
| India | 0 |  | 0.0023 | -0.3921 | -0.3628 | -0.3148 |
|  | 1 | 0.308 | 0.0025 | -0.3269 | -0.239 | -0.0953 |
|  | 2 | 0.842 | 0.0028 | -0.1925 | -0.0461 | 0.1936 |
|  | 3 | 0.155 | 0.0029 | -0.1651 | 0.04 | 0.3754 |
|  | 4 | 0.731 | 0.0033 | -0.0432 | 0.2205 | 0.6518 |
|  | 5 | 0 | 0.0014 | -0.9101 | -0.5879 | -0.0607 |
|  | 6 | 0 | 0.0008 | -1.5405 | -1.15962* | -.536641* |
|  | 7 | 0.031 | 0.0007 | -1.594 | -1.1546 | -0.4358 |
|  | 8 | 0.029 | .000698* | -1.65091* | -1.1529 | -0.3382 |
|  | 9 | 0.865 | 0.0008 | -1.5137 | -0.9571 | -0.0466 |
|  | 10 | 0.229 | 0.0009 | -1.4653 | -0.8501 | 0.1563 |
| Indonesia | 0 |  | .006216* | .595045* | .624341* | .672262* |
|  | 1 | 0.458 | 0.0068 | 0.6842 | 0.7721 | 0.9158 |
|  | 2 | 0.617 | 0.0076 | 0.7933 | 0.9398 | 1.1794 |
|  | 3 | 0.384 | 0.0082 | 0.8716 | 1.0767 | 1.4121 |
|  | 4 | 0.895 | 0.0095 | 1.0125 | 1.2762 | 1.7075 |
|  | 5 | 0.017 | 0.0088 | 0.93 | 1.2523 | 1.7794 |
|  | 6 | 0.856 | 0.0102 | 1.0661 | 1.447 | 2.0699 |
|  | 7 | 0.94 | 0.012 | 1.2133 | 1.6527 | 2.3715 |
|  | 8 | 0 | 0.0093 | 0.94 | 1.438 | 2.2527 |
|  | 9 | 0.062 | 0.0094 | 0.92 | 1.4766 | 2.3871 |
|  | 10 | 0.293 | 0.0103 | 0.9823 | 1.5976 | 2.6039 |
| Iran | 0 |  | 0.0196 | 1.7441 | 1.7734 | 1.82133* |
|  | 1 | 0.025 | .018397* | 1.67987* | 1.76775* | 1.9115 |
|  | 2 | 0.64 | 0.0206 | 1.7916 | 1.9381 | 2.1777 |
|  | 3 | 0.984 | 0.0241 | 1.9471 | 2.1522 | 2.4877 |
|  | 4 | 0.887 | 0.0279 | 2.087 | 2.3506 | 2.7819 |
|  | 5 | 0.042 | 0.027 | 2.0485 | 2.3708 | 2.8979 |
|  | 6 | 0.022 | 0.0254 | 1.9786 | 2.3594 | 2.9824 |
|  | 7 | 0.874 | 0.0296 | 2.1169 | 2.5563 | 3.2751 |
|  | 8 | 0.795 | 0.0344 | 2.2459 | 2.7439 | 3.5586 |
|  | 9 | 0.742 | 0.0399 | 2.3691 | 2.9257 | 3.8362 |
|  | 10 | 0.055 | 0.0402 | 2.3434 | 2.9587 | 3.965 |
| Japan | 0 |  | 0.0069 | 0.69996 | 0.72926 | 0.77718 |
|  | 1 | 0 | 0.0042 | 0.20217 | .290059* | .433823* |
|  | 2 | 0.052 | .004086* | .174192* | 0.32067 | 0.56028 |
|  | 3 | 0.62 | 0.00457 | 0.28355 | 0.48863 | 0.82407 |
|  | 4 | 0.463 | 0.00502 | 0.37338 | 0.63705 | 1.06833 |
|  | 5 | 0.406 | 0.00549 | 0.45499 | 0.77725 | 1.30438 |
|  | 6 | 0.086 | 0.00553 | 0.45165 | 0.83249 | 1.45547 |
|  | 7 | 0.596 | 0.00624 | 0.55823 | 0.99767 | 1.71649 |
|  | 8 | 0.308 | 0.00679 | 0.62354 | 1.12157 | 1.93623 |
|  | 9 | 0.025 | 0.00653 | 0.55869 | 1.11531 | 2.02581 |
|  | 10 | 0.122 | 0.00685 | 0.57341 | 1.18863 | 2.19497 |
| Korea | 0 |  | 0.0086 | 0.9185 | 0.9478 | 0.9957 |
|  | 1 | 0 | .006415* | .626253* | .714142* | .857905* |
|  | 2 | 0.863 | 0.0074 | 0.7632 | 0.9097 | 1.1493 |
|  | 3 | 0.148 | 0.0076 | 0.7882 | 0.9933 | 1.3287 |
|  | 4 | 0.879 | 0.0087 | 0.9271 | 1.1907 | 1.622 |
|  | 5 | 0.008 | 0.0078 | 0.8075 | 1.1298 | 1.6569 |
|  | 6 | 0.586 | 0.0088 | 0.9129 | 1.2938 | 1.9168 |
|  | 7 | 0.68 | 0.01 | 1.0292 | 1.4686 | 2.1874 |
|  | 8 | 0.904 | 0.0117 | 1.1713 | 1.6693 | 2.484 |
|  | 9 | 0.347 | 0.013 | 1.2435 | 1.8001 | 2.7106 |
|  | 10 | 0.003 | 0.0114 | 1.0813 | 1.6965 | 2.7029 |
| Malaysia | 0 |  | .007179* | .739104* | .7684* | .816321* |
|  | 1 | 0.33 | 0.0077 | 0.8084 | 0.8962 | 1.04 |
|  | 2 | 0.178 | 0.008 | 0.8432 | 0.9897 | 1.2293 |
|  | 3 | 0.124 | 0.0081 | 0.8589 | 1.064 | 1.3995 |
|  | 4 | 0.96 | 0.0095 | 1.0095 | 1.2731 | 1.7044 |
|  | 5 | 0.115 | 0.0097 | 1.0214 | 1.3436 | 1.8708 |
|  | 6 | 0.027 | 0.0092 | 0.9616 | 1.3425 | 1.9654 |
|  | 7 | 0.814 | 0.0106 | 1.0929 | 1.5323 | 2.2511 |
|  | 8 | 0.573 | 0.012 | 1.1967 | 1.6947 | 2.5094 |
|  | 9 | 0.005 | 0.0107 | 1.0528 | 1.6094 | 2.5199 |
|  | 10 | 0.363 | 0.0119 | 1.1277 | 1.7429 | 2.7493 |
| Myanmar | 0 |  | 0.0059 | 0.536 | 0.5652 | 0.6132 |
|  | 1 | 0.6 | 0.0065 | 0.643 | 0.7309 | 0.8746 |
|  | 2 | 0.99 | 0.0076 | 0.8001 | 0.9465 | 1.1862 |
|  | 3 | 0.711 | 0.0086 | 0.9198 | 1.1249 | 1.4603 |
|  | 4 | 0.215 | 0.0091 | 0.9647 | 1.2284 | 1.6596 |
|  | 5 | 0 | 0.0025 | -0.3414 | -0.0191 | 0.508 |
|  | 6 | 0 | 0.0005 | -1.8725 | -1.49166* | -.86869* |
|  | 7 | 0.033 | .000521* | -1.9239 | -1.4844 | -0.7656 |
|  | 8 | 0.426 | 0.0006 | -1.8393 | -1.3413 | -0.5266 |
|  | 9 | 0.014 | 0.0005 | -1.93275* | -1.3761 | -0.4656 |
|  | 10 | 0.538 | 0.0006 | -1.8332 | -1.218 | -0.2117 |
| Nepal | 0 |  | 0.00248 | -0.325 | -.295669* | -.247748* |
|  | 1 | 0.245 | 0.00261 | -0.2728 | -0.1849 | -0.0412 |
|  | 2 | 0.331 | 0.0028 | -0.2035 | -0.057 | 0.18264 |
|  | 3 | 0.163 | 0.00289 | -0.1734 | 0.03168 | 0.36713 |
|  | 4 | 0.133 | 0.00296 | -0.1539 | 0.10973 | 0.54102 |
|  | 5 | 0 | .00207* | -.52024* | -0.198 | 0.32915 |
|  | 6 | 0.845 | 0.00239 | -0.3855 | -0.0047 | 0.61833 |
|  | 7 | 0.343 | 0.00261 | -0.314 | 0.12545 | 0.84427 |
|  | 8 | 0.279 | 0.00282 | -0.2544 | 0.24362 | 1.05828 |
|  | 9 | 0.281 | 0.00307 | -0.1945 | 0.36215 | 1.27265 |
|  | 10 | 0.069 | 0.00313 | -0.2086 | 0.4066 | 1.41294 |
| Pakistan | 0 |  | 0.0005 | -1.9692 | -1.9401 | -1.8927 |
|  | 1 | 0 | 0.0002 | -3.0728 | -2.9854* | -2.84333* |
|  | 2 | 0.141 | 0.0002 | -3.0509 | -2.9052 | -2.6685 |
|  | 3 | 0.327 | 0.0002 | -2.9835 | -2.7796 | -2.4482 |
|  | 4 | 0.838 | 0.0002 | -2.8522 | -2.5901 | -2.1639 |
|  | 5 | 0.825 | 0.0002 | -2.7224 | -2.4021 | -1.8811 |
|  | 6 | 0.006 | 0.0002 | -2.849 | -2.4704 | -1.8548 |
|  | 7 | 0 | .000142* | -3.22122* | -2.7844 | -2.074 |
|  | 8 | 0.281 | 0.0002 | -3.1625 | -2.6674 | -1.8624 |
|  | 9 | 0.511 | 0.0002 | -3.0683 | -2.5149 | -1.6151 |
|  | 10 | 0.18 | 0.0002 | -3.0335 | -2.4219 | -1.4274 |
| Philippines | 0 |  | .004632* | .301071* | .330367* | .378288* |
|  | 1 | 0.129 | 0.0047 | 0.3186 | 0.4065 | 0.5503 |
|  | 2 | 0.724 | 0.0053 | 0.4398 | 0.5862 | 0.8258 |
|  | 3 | 0.997 | 0.0063 | 0.5996 | 0.8047 | 1.1401 |
|  | 4 | 0.922 | 0.0073 | 0.7441 | 1.0078 | 1.4391 |
|  | 5 | 0.198 | 0.0076 | 0.7846 | 1.1069 | 1.634 |
|  | 6 | 0.379 | 0.0083 | 0.8621 | 1.243 | 1.866 |
|  | 7 | 0.672 | 0.0095 | 0.9775 | 1.4169 | 2.1357 |
|  | 8 | 0.849 | 0.0111 | 1.1127 | 1.6108 | 2.4254 |
|  | 9 | 0.001 | 0.0093 | 0.9073 | 1.4639 | 2.3744 |
|  | 10 | 0.002 | 0.0079 | 0.7132 | 1.3285 | 2.3348 |
| Singapore | 0 |  | 0.09643 | 3.33685 | 3.36614* | 3.41406* |
|  | 1 | 0.232 | 0.10133 | 3.38604 | 3.47393 | 3.61769 |
|  | 2 | 0.017 | .093514* | 3.30469 | 3.45117 | 3.69077 |
|  | 3 | 0.131 | 0.0955 | 3.32316 | 3.52823 | 3.86368 |
|  | 4 | 0.583 | 0.10656 | 3.42827 | 3.69193 | 4.12322 |
|  | 5 | 0.009 | 0.09575 | 3.31418 | 3.63643 | 4.16357 |
|  | 6 | 0.138 | 0.09883 | 3.33534 | 3.71619 | 4.33916 |
|  | 7 | 0.029 | 0.09474 | 3.27853* | 3.71797 | 4.43678 |
|  | 8 | 0.776 | 0.10968 | 3.40549 | 3.90352 | 4.71818 |
|  | 9 | 0.144 | 0.11518 | 3.42888 | 3.98551 | 4.89601 |
|  | 10 | 0.04 | 0.11412 | 3.38693 | 4.00215 | 5.00849 |
| Sri Lanka | 0 |  | 0.0061 | 0.5759 | 0.6052 | .653105* |
|  | 1 | 0.006 | .005352* | .445106* | .532994* | 0.6768 |
|  | 2 | 0.683 | 0.006 | 0.5617 | 0.7082 | 0.9478 |
|  | 3 | 0.956 | 0.007 | 0.7115 | 0.9166 | 1.2521 |
|  | 4 | 0.645 | 0.0079 | 0.8238 | 1.0875 | 1.5188 |
|  | 5 | 0.06 | 0.0078 | 0.8023 | 1.1245 | 1.6517 |
|  | 6 | 0.638 | 0.0088 | 0.9138 | 1.2946 | 1.9176 |
|  | 7 | 0.859 | 0.0102 | 1.0502 | 1.4897 | 2.2085 |
|  | 8 | 0.015 | 0.0095 | 0.962 | 1.4601 | 2.2747 |
|  | 9 | 0.384 | 0.0106 | 1.0402 | 1.5968 | 2.5073 |
|  | 10 | 0 | 0.0081 | 0.7361 | 1.3513 | 2.3576 |
| Thailand | 0 |  | 0.0029 | -0.1716 | -0.1423 | -0.0944 |
|  | 1 | 0 | .001361* | -.924003* | -.836115* | -.692352* |
|  | 2 | 0.651 | 0.0015 | -0.811 | -0.6645 | -0.4249 |
|  | 3 | 0.67 | 0.0017 | -0.6958 | -0.4908 | -0.1553 |
|  | 4 | 0.292 | 0.0018 | -0.6338 | -0.3701 | 0.0612 |
|  | 5 | 0.359 | 0.002 | -0.5595 | -0.2372 | 0.2899 |
|  | 6 | 0.81 | 0.0023 | -0.4287 | -0.0478 | 0.5752 |
|  | 7 | 0.785 | 0.0026 | -0.3007 | 0.1388 | 0.8576 |
|  | 8 | 0.398 | 0.0029 | -0.2203 | 0.2777 | 1.0924 |
|  | 9 | 0.185 | 0.0031 | -0.1835 | 0.3732 | 1.2837 |
|  | 10 | 0.971 | 0.0037 | -0.031 | 0.5843 | 1.5906 |
